# Perceptions and Determinants of Eating for Health and Performance in High-Level Male Adolescent Rugby Union Players

**DOI:** 10.3390/sports6020049

**Published:** 2018-05-28

**Authors:** Emily G. Stokes, Roger Hughes, David M. Shaw, Helen T. O’Connor, Kathryn L. Beck

**Affiliations:** 1 School of Sport Exercise and Nutrition, College of Health, Massey University, Auckland 0745, New Zealand; emgracestokes@gmail.com; 2 School of Medicine, University of Tasmania, Hobart 7001, Australia; roger.hughes@utas.edu.au; 3 The Blues Rugby Franchise, Auckland 1051, New Zealand; nutrition@daveshaw.co.nz; 4 Discipline of Exercise and Sport Science, Faculty of Health Sciences and Charles Perkins Centre, The University of Sydney, Sydney 1825, Australia; helen.oconnor@sydney.edu.au

**Keywords:** athlete, barrier, diet, nutrition, qualitative, sport

## Abstract

Sports nutrition recommendations provide guidance on dietary strategies to optimise sports performance. However, research indicates that young athletes often find it difficult to follow these guidelines in practice. Limited research exists on the determinants that influence adherence to sports nutrition guidelines. This study aimed to explore the perceptions and determinants of eating for health and performance in high-level male adolescent rugby union players. Determinants were explored using semi-structured individual interviews in New Zealand high-level male rugby union players (*n* = 20, 16–18 years). Interviews were recorded, transcribed, and then underwent thematic analysis. Perceptions of eating for health and performance included balance and variety, appropriate portions, and specific foods. Both adolescent- and sport-specific determinants influenced the food choices of participants. Determinants relevant to adolescent lifestyles included the influence of significant others such as peers and family but also included the taste, cost, convenience, and availability of food. Sports-specific determinants revolved around the desire to enhance sports performance, motivation to perform, and team culture. The media (mainstream and social media), physical appearance, and feeling good were identified as both adolescent- and sport-specific factors influencing food choice. These findings highlight the importance of having support and positive role modelling to help young athletes make optimal food choices for health and performance. Strategies to further enable healthy eating practices should aim to strengthen the support available to young athletes in the home, school, and sporting environments and should include education on appropriate social media use to inform eating for health and performance.

## 1. Introduction

Application of evidence-based sports nutrition principles is central for optimising sports performance and supporting growth and lean mass development in adolescent rugby union (RU) players. Although robust scientific position stands [1,2], and evidence-based resources exist to guide young players on optimal dietary strategies [3,4], the dietary intake of adolescent RU players appears to fall short of these guidelines [5,6], including inadequate fruit and vegetables and high intakes of discretionary (treat) foods [6]. Similar findings are reported in other adolescent sporting groups [7,8]. Although nutrition education may support improved dietary intakes, knowledge does not always translate into practice [9]. Therefore, exploring enablers and barriers to food choice through determinants analysis is valuable for informing a targeted strategy to support improved adherence to sports nutrition recommendations.

Numerous studies have explored determinants of healthy eating in adolescent populations [10]. Factors commonly identified as healthy eating enablers include family support, access to healthy food, and goals related to appearance [10]. Commonly reported barriers include limited healthy food availability, the perception that healthy food is more expensive, and the taste and convenience of less healthy food choices [10]. These factors are likely to influence the food choice of adolescent athletes, although additional determinants related to performance are likely to be important [11].

Few studies have investigated determinants of eating for health and performance in athletes [11]. A lack of time [12] and food cost have been reported as barriers to healthy eating for athletes, along with hedonic hunger, taste preferences, and reduced motivation to eat well during off-season [11,12].

Given the limited body of research exploring eating determinants in adolescent athletes and the value of obtaining a deeper insight into factors influencing their food choices, this study aimed to explore perceptions and determinants of eating for health and performance in high-level male adolescent RU players. Perceptions were classified as what participants thought eating for health and performance meant and determinants as factors influencing participants’ food choices with regards to eating for health and performance.

## 2. Materials and Methods

Twenty male adolescent RU players aged 16–18 years (17 ± 1 years; height: 182 ± 9 cm; weight: 97 ± 12 kg; body mass index (BMI): 29 ± 5 kg/m^2^) were recruited from four secondary schools, five participants each, from a range of socio-demographic regions across Auckland, New Zealand. The sports manager and/or rugby coach from the top rugby team from each school facilitated access to the most talented players. Ethical approval was granted by the Massey University Human Ethics Committee Northern (reference NOR 16/33) and participants provided informed written consent. All invited players agreed to participate.

This study employed a cross-sectional qualitative design. Participants completed an online questionnaire, developed by the research team, regarding demographics, self-reported height and weight, training regimen (type and hours per week), and dietary supplement intake. A trained researcher (ES) undertook individual face-to-face semi-structured interviews with participants on school grounds to explore perceptions and determinants of eating for health and performance.

All interviews were audio recorded and lasted 40 to 60 min. The interview schedule (Table 1) developed by the research team included questions related to perceptions of a healthy and unhealthy diet, enablers and barriers to eating healthy, and the perceived impact of dietary intake on health and performance. Care was taken not to lead participant responses. The researcher probed participants to enhance clarity and assist in elaboration of participants’ ideas. Thematic saturation was achieved (i.e., further interviews were unlikely to elicit new information) [13].

Interviews were transcribed verbatim by one researcher (ES), with thematic analysis used to explore key ideas. Initial coding and theme development was undertaken manually by one researcher (ES). Two researchers (KB, RH) read all transcripts and confirmation and consensus regarding themes was achieved through discussion (ES, KB, RH). Quotes were extracted to represent the themes identified, with selected verbatim quotes presented in the results. Participant characteristics were described using mean ± standard deviation (SD) for continuous and *n* (%) for categorical data.

## 3. Results

### 3.1. Participant Characteristics

Participant ethnicities were 35% New Zealand European, 35% Samoan, 20% Tongan, and 10% Māori. On average, participants trained 14.4 ± 8 h per week. The highest representative level for most players (60%) was regional, followed by school (25%), national (10%), and international/age group (5%). Fifty percent of participants took dietary supplements including sports drinks (30%), vitamin/minerals (25%), sports bars (15%), protein powder (15%), pre-workout supplements (5%), and creatine monohydrate (5%).

### 3.2. Perceptions of Eating for Health and Performance

The key themes depicting perceptions of eating for health and performance included balance and variety, appropriate portions, and specific foods. A common response for a healthy diet was to obtain variety and a balance of carbohydrates, protein, and fruit and vegetables (65% of participants). Having the ‘right amount’ of food was associated with a healthy diet for 40% of participants. This meant not over-eating, especially before training and games.

“*If you don’t have a balanced diet … you get too much energy and then you don’t burn it off and then that turns into fat—so that’s kind of unhealthy.*”

Participant 2

“*Taking the right amount, you don’t want to be going over [eating too much], especially if you have training that day … [could lead to] getting stitch.*”

Participant 8

Specific foods perceived as healthy were mentioned by all participants. These foods included fruit, vegetables, meat, fish, eggs, milk, yoghurt, porridge, wheat biscuits, rice, pasta, bread, and water. Protein supplements and sports drinks were described as healthy by three participants. However, 75% of participants thought they were too young to take performance-enhancing supplements, perceiving natural sources of protein as better and sports drinks as only beneficial during exercise.

“*You can only drink [sports drinks] when you are training hard or after a hard session.*”

Participant 10

“*I feel protein bars are good but artificial, when you have real meals it’s better and it’s [protein bars] a bit of a lazy choice.*”

Participant 5

Foods mentioned as unhealthy were takeaway foods including pies, pizza, fried food, and potato chips (85% of participants); sweets including cake, chocolate, ice-cream, lollies (90% of participants); and soft drinks (50% of participants). These foods were described as high in fat, sugar, and calories and processed.

“*[Why such foods are unhealthy] full of fat, fat’s good in moderation but when it’s high … Full of sugar, if the sugar has been added then to me it’s not that great.*”

Participant 1

### 3.3. Determinants of Eating for Health and Performance

**Figure 1:**
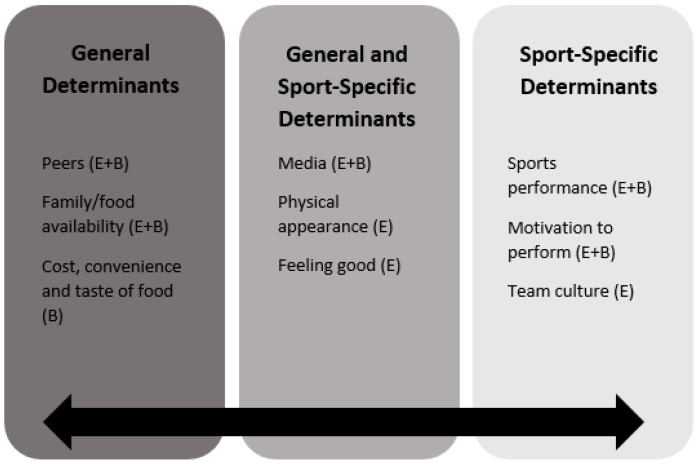
Diagram depicting the main themes of eating for health and performance as general, sport-specific, and both sport-specific and general determinants. E = enabler, B = barrier.

Participants identified several barriers and enablers to eating healthy. The main themes were classified into general, sport-specific, and both general and sport-specific determinants (Figure 1).

#### 3.3.1. General Determinants

Peers were mentioned by 55% of participants as enablers to healthy eating when they were supportive and ate healthy, for example, by bringing healthy options to school and telling participants not to choose unhealthy foods. A few participants and friends were competitive with healthy eating.

“*I guess they kind of motivate me, because for our lunches we try and make a proper meal. We are quite competitive—always got to have the best lunch.*”

Participant 2

Peers were a barrier to healthy eating (75% of participants) when they ate unhealthy food, as this provided temptation.

“*I have a lot of mates who don’t play sport and they bring their own food from home that’s unhealthy. It’s hard to try and resist.*”

Participant 9

Family was an enabler (80% of participants) and barrier (35%) to healthy eating based on food provision. Family enabled healthy eating when they reminded participants that unhealthy eating is not good for the body, and by role modelling healthy eating, particularly family members who played professional rugby. Siblings provided temptation to eat unhealthily.

“*My family in general, we are quite a healthy eating sort of family. So obviously if they’re eating healthy, you’re eating healthy because they are preparing all the meals.*”

Participant 1

*“… I’ve lived a lot with my uncle and while I was with him he played in the Warriors [professional rugby team] … I noticed he would cook his own meals. He wouldn’t buy fast foods … being around him gave me the drive to do the same thing.*”

Participant 13

For the two participants who boarded at school, the food provided was not always healthy and meals were not always available before or after training.

“*After breakfast there isn’t another meal until lunch so if you don’t eat straight after training you can’t get breakfast.*”

Participant 13

Barriers to healthy eating were the cost of food (50% of participants), taste preferences for unhealthy food (65% of participants), and the time taken to prepare healthy meals (40% of participants), especially when energy levels were low, for example, after school and training.

“*Probably having to make it [healthy food] yourself. Sometimes I am just too tired.*”

Participant 3

#### 3.3.2. General and Sport-Specific Determinants

One-third of participants described media (television, internet, and social media) as an enabler and barrier to healthy eating. Participants were motivated to eat healthy when media showed the negative outcomes of unhealthy eating. Advertisements that displayed unhealthy food in an appealing way created temptation.

“*Advertisements—you compare how much calories and fat there is. I was just disgusted—you see it on Facebook.*”

Participant 4

A few participants followed their role models on social media to obtain inspiration and motivation to eat healthy by observing what they ate. Information on healthy eating was sourced using Google search.

“*I saw one of the All Blacks has eggs on toast before a game, so I thought that would be good, so I try to have eggs, toast for breakfast on game day and water.*”

Participant 5

Physical appearance was a motivating factor for 25% of participants to eat healthy to maintain or lose weight, gain muscle mass, or look good in general. Eating unhealthily was linked with unwanted weight gain.

“*[benefit of healthy eating] keep a good weight … these benefits push me to do better and eat healthier.*”

Participant 18

Healthy eating was associated with feeling better for 80% of participants—physically through maintaining a desired physique, fitness, and energy. This made players feel positive—including increased confidence, happiness, and focus, leading to improved performance. Unhealthy eating was associated with feeling lazy.

“*You just feel more happy and energetic, don’t feel sluggish [with healthy eating].*”

Participant 11

#### 3.3.3. Sport-Specific Determinants

Knowing the benefit healthy eating had on performance (for example, increased energy and better recovery) influenced food choices for all participants, especially around game day. Nearly half of the participants (45%) expressed the importance of allowing sufficient time for food to digest before playing rugby, as well as choosing appropriate portions and types of food that would not upset the stomach during the game. Unhealthy foods, such as takeout foods, made participants feel weighed down, while eating too little led to not having enough energy.

“*Before a game, if I was to eat till I’m full I’d feel heavy, sluggish. If I had it [meal] right before a game, my body has to have time to digest.*”

Participant 5

Personal motivation influenced 80% of participants, knowing that healthy eating would help accomplish their performance goals. Three participants described their motivation as reduced when the rugby season was finished or if injured.

“*If you get a major injury, you’ve kind of got nothing to do. For me I found, oh I’m just going to pass time, so I think that stopped me from my eating healthy plan I wanted to stick to.*”

Participant 1

Team culture (including coaches, trainers, sports management, and teammates) was described by 60% of participants as an enabler to healthy eating through the provision of advice and encouragement on healthy eating.

“*I feel our team culture has been pretty good this year and we all keep each other on track. As a team, we all remind each other [to eat healthy].*”

Participant 5

## 4. Discussion

In this study, perceptions of healthy eating were generally in accordance with New Zealand healthy eating guidelines [14]. Participants described healthy eating as having balance and variety, appropriate portions, and specific foods. In New Zealand, the distribution of carbohydrates, protein, and fruit and vegetables (balance) are commonly described as the ‘healthy plate model’, which many participants would have encountered at school and during rugby nutrition talks. Other studies in adolescents have also described balance, variety [15,16], and appropriate portions [15] as important within a healthy diet. Similar to our findings, fruit and vegetables are generally considered by adolescents as healthy, whilst additional foods perceived as healthy vary [15,16,17,18,19,20]. Unhealthy foods specified in our study (takeaway foods, sugary foods, soft drinks) were similar to those specified by adolescents [10,15,16,19,21] and athletes [12,22] in other studies.

Sports drinks and protein supplements were described by three participants as part of eating for health and performance. While natural food sources of protein were preferred, protein supplements (e.g., protein bars) were described as convenient, as they were pre-prepared and saved time as well as being perceived as providing greater amounts of protein than traditional foods. Three participants reported taking protein powders, which is less than in previous studies of adolescent rugby players [5,23]. The participants’ position of natural food being better than supplements is in line with the Sports Dietitians Australia recommendation that nutrition requirements are met by food rather than supplements [1].

Determinants of eating for health and performance related to participants’ general lifestyles as adolescents and sporting lifestyles as rugby players. General determinants were peers, family, and food availability, and taste, cost, and convenience of food. Other studies in adolescents found lack of peer support [24] and peer pressure [20] as barriers, while peer approval for healthy eating facilitated healthy eating [25]. In this study, peers tempted rather than pressured participants to eat unhealthily.

The provision of healthy food at home and healthy eating role modelling from parents helped participants to eat healthy. For American adolescents, food availability at home and parental role modelling also facilitated healthy eating [26]. Male hockey players reported family influencing their eating practices at home [22]. Educating peers and family on creating supportive environments for healthy eating could assist adolescent athletes to eat healthily.

The taste of unhealthy food appealed to participants, which has been widely established [10,15,16,17,18,20]. Male hockey players reported fast foods and sweets as tasting good [22]. Affordability and convenience are frequently reported by adolescents as determinants of food choice [10,15,17,18,20,27]. In this study, participants described themselves as lacking energy after school and training, therefore opting for convenience foods, as well as perceiving less healthy foods to be the cheaper option. Australian athletes also perceived unhealthy food to be convenient and cheaper [12]. Affordable, tasty, and convenient healthy food ideas should be provided to young athletes, for example, fast food alternatives or recipes.

Determinants overlapping general and sporting lifestyles were media, physical appearance, and feeling good. Professional rugby players on social media inspired participants to eat similar food. Advertisements created temptation for unhealthy eating or motivated healthy eating when negative effects of unhealthy eating were shown. Other studies have reported advertising but not social media as influencing adolescent’s food choice [16,27]. An increase in social media use may explain differences in findings [28]. Ensuring adolescent athletes understand the marketing associated with media is key to supporting them develop a healthy mind set around food.

The impact of healthy eating on physical appearance and feeling good motivated participants to eat healthy. Female athletes have linked unhealthy eating to weight gain [12] and the ‘feel good factor’ of healthy eating has been reported in Australian adolescents, making them feel revived [20]. These factors could be emphasised in education sessions as a motivator for eating healthy.

Sport-specific determinants were sports performance, motivation to perform, and team culture. Knowing the impact food choices have on sports performance motivated participants to eat healthy. However, during off-season or injury, motivation to eat healthy reduced. Temptation to eat unhealthily during ‘off-season’ has been reported by male hockey players [22]. Creating strategies, such as continued contact through ‘off-season’ or injury, is important to keep adolescent athletes motivated to eat healthy. Unhealthy and large amounts of foods were avoided close to the game, as they made participants feel sluggish. Athletes have previously reported gastrointestinal discomfort influencing food choices around competition [29]. Appropriate food choices should be available on game day to maximise sporting performance.

Team culture was a positive influence on healthy eating, with teammates and coaching staff a source of encouragement and nutrition advice. Male collegiate football players reported teammates who ate healthy influenced them to also eat healthy [30]. Athletes may follow the eating practices of their teammates due to wanting to comply with what is socially acceptable [31]. Creating a positive team environment will further enable adolescent athletes to eat healthy. As coaches and trainers are a trusted source of nutrition information, it is essential that they are educated on healthy eating practices. Table 2 provides a summary of recommendations based on the results of this study for professionals working with adolescent athletes to optimise eating for health and performance.

Strengths and limitations must be considered when interpreting the results of this study. Participants were from schools representing a range of ethnicities and socio-demographic backgrounds. However, schools recruited were all in Auckland, with access to nutritionists, limiting the generalisability of results. Further research should explore determinants of healthy eating in other adolescent athletes and whether these determinants are associated with dietary intake. The advantage of undertaking individual interviews is that this format excludes the potential of peer influence, which may occur with focus groups, therefore, participants may have felt more comfortable expressing their views. However, participants may not have been completely honest in order to please the interviewer and meet perceived expectations. Investigator triangulation provided greater truthfulness and credibility to findings due to the cross-verification process used to explore and articulate themes.

## 5. Conclusions

In conclusion, high-level male adolescent rugby players living in New Zealand have a good understanding of what eating for health and performance means. Determinants of healthy eating related to both adolescent- and sport-related lifestyles. These factors should be considered when developing strategies to support adolescent rugby players to eat optimally for health and performance in the home, school, and sports environment.

## Figures and Tables

**Table 1:** Questions used to promote discussion in the interviews.

Describe in your own words what you think a healthy diet is
Why do you think this is healthy? Can you provide some examples of healthy foods?
Describe in your own words what you think an unhealthy diet is
Why do you think this is unhealthy? Can you provide some examples of unhealthy foods?
What do you think are the benefits of healthy eating?
Why do you think that? Do these benefits influence what you eat?
What makes it easier for you to eat healthy?
What gets in the way/makes it more difficult for you to eat healthy?
Do you think that what you eat has an influence on how you perform?
No: Why? Yes: What types of foods do you think have an influence on your rugby playing? Why?
Are there any specific foods, drinks, or products that you can take to help your rugby playing?
No: Why? Yes: How do you think that works? What benefits do they bring?
Is there anything we haven’t talked about that affects what you eat?

**Table 2:** Recommendations for professionals working with adolescent athletes to optimise eating for health and performance.

Educate family and peers on the importance of creating supportive environments for healthy eating at home and school.
Provide affordable, tasty, and convenient healthy food ideas to athletes, for example, fast food alternatives or recipes.
Ensure athletes understand the limitations associated with the media and social media (e.g., Facebook, Instagram) as a source of nutrition advice.
Emphasise how healthy eating makes athletes feel and link food choices to performance in education sessions as a motivator for eating for health and performance.
Maintain contact with players through the ‘off-season’ or injury to help athletes stay motivated to eat for health and performance.
Provide appropriate food choices on game day to maximise sporting performance and prevent gastrointestinal discomfort.
Create a positive team environment towards eating for health and performance.
Educate coaches and trainers on eating practices that are safe and in line with evidence-based sports nutrition guidelines.
